# Determination of *Enterococcus faecalis* and *Enterococcus faecium* Antimicrobial Resistance and Virulence Factors and Their Association with Clinical and Demographic Factors in Kenya

**DOI:** 10.1155/2022/3129439

**Published:** 2022-11-09

**Authors:** Martin Georges, Erick Odoyo, Daniel Matano, Fredrick Tiria, Cecilia Kyany'a, Daniel Mbwika, Winnie C. Mutai, Lillian Musila

**Affiliations:** ^1^Department of Emerging Infectious Diseases, United States Army Medical Research Directorate-Africa, Nairobi, Kenya; ^2^Kenya Medical Research Institute, Nairobi, Kenya; ^3^Kenyatta University, Nairobi, Kenya; ^4^Department of Medical Microbiology, School of Medicine, University of Nairobi, Nairobi, Kenya

## Abstract

**Background:**

Enterococci are clinically significant because of their increasing antibiotic resistance and their ability to cause severe infections due to an arsenal of virulence genes. Few studies in the developing world have examined virulence factors that may significantly impact patient outcomes. This study describes the antimicrobial resistance profiles and prevalence of five key *Enterococcal virulence* genes *gelE*, *asa*, *cylA*, *esp,* and *hyl* in forty-four clinical *Enterococcus faecalis* and *E. faecium* isolates in Kenya and their association with patients' demographic and clinical characteristics.

**Results:**

All *E. faecium* isolates were obtained from hospital-acquired skin and soft tissue infections. While *E. faecalis* was associated with community-acquired urinary tract infections. All isolates were resistant to erythromycin, whereas 11/44 (27.5%), 25/44 (56.8%), 28/44 (63.6%), 37/44 (84.1%), 40/44 (90.0%), and 43/44 (97.5%) were susceptible to tetracycline, levofloxacin, gentamicin, ampicillin, nitrofurantoin, and teicoplanin, respectively. All isolates were susceptible to tigecycline, vancomycin, and linezolid. There was little difference in the antibiotic resistance profiles between *E. faecalis* and *E. faecium*. The prevalence of the virulence genes among the 44 isolates were 27 (61.4%) for *gelE*, 26 (59.1%) for *asa1*, 16 (36.3%) for *esp,* 11 (25.0%) for *cylA,* and 1 (2.3%) for *hyl*. 72.9% of *E. faecalis* isolates had multiple virulence genes compared to 57% of *E. faecium* isolates with no virulence genes. The *hyl* gene was only detected in *E. faecium,* while *cylA* and *asa1* were only detected in *E. faecalis*. A significant correlation was observed between the presence of *asa1* and *esp* virulence genes and tetracycline resistance (*P*=0.0305 and 0.0363, respectively). A significant correlation was also observed between the presence of virulence genes *gelE* and *asa1* and nitrofurantoin resistance (*P*=0.0175 and 0.0225, respectively) and ampicillin resistance (*P*=0.0005 and 0.0008, respectively).

**Conclusion:**

The study highlights the high levels of erythromycin resistance in *E. faecalis* and *E. faecium*, the demographic factors influencing the species distribution among patients, and the accumulation of multiple virulence genes in *E. faecalis.* The significant association of *gelE*, *asa1,* and *esp* virulence genes with drug resistance could explain the pathogenic success of *E. faecalis* and provides a guide for future studies.

## 1. Background

Enterococcal species bacteria are Gram-positive cocci typically found in the gut, bowel, throat, mouth, and vagina as commensals [1–4]. *E. faecalis* and *E. faecium* are the main pathogenic species of the eighteen-known species of enterococci. However, only the *E. faecalis* and *E. faecium* strains harboring virulence genes are associated with human infections [5], including urinary tract (UTI), pelvic, blood, intraabdominal, and skin and soft tissue infections (SSTI) [6]. The main virulence factors that have been described in Enterococci are aggregation substance (*asa1*), gelatinase (*gelE*), cytolysin (*cylA*), enterococcal surface protein (*esp*), and hyaluronidase (*hyl*). *asa1* is an aggregation substance that enhances adherence to renal tubular cells [1]. Gelatinase is a zinc metalloprotease that hydrolyzes fibrin, collagen, and other peptides [7] and has been linked to biofilm formation [8]. The secretion of hemolysins such as cytolysin causes the breakdown of blood cells and has been linked with increased toxicity in human infection [9]. Hyaluronidase facilitates the colonization of host tissue by breaking down hyaluronic acid, a critical component of connective tissue [10]. Enterococcal surface protein (*esp*) is a high molecular weight surface protein associated with biofilm formation that is specific to enterococci [11]. *esp* also plays a role in colonization and persistence in the urinary tract [12]. The enterococcal virulence gene *gelE* is the most common in *E. faecalis* isolates, while *hyl* and *esp* are more common in *E. faecium* than in *E. faecalis* [13]. Most of what is known about Enterococci species virulence is based on studies outside Africa [14, 15].

There is limited published data on the prevalence and distribution of virulence genes among clinical enterococci in Africa. However, studies in Ethiopia have demonstrated that Enterococcal isolates from animals carry the *gelE* virulence genes [16], contribute to hospital-associated infections [17] and profiled their antimicrobial susceptibility [18]. Enterococci species are not major clinical pathogens in Africa as reports in Nigeria and Kenya indicate a point prevalence of 5–11% [19]. Despite this, resistance to glycopeptides, aminoglycosides, *β*-lactams, and fluoroquinolone antibiotic classes is on the rise globally [10, 20] and could pose challenge to the treatment of these infections given that the remaining treatment options, such as linezolid, daptomycin quinupristin/dalfopristin, and vancomycin, are expensive with limited availability in primary care centers in Kenya. Although rare, there have been reports of vancomycin resistance in Kenya [19], indicating the possibility of growing resistance to these last-line drugs. The interplay of virulence genes and antimicrobial resistance in clinical infections is worth examining as hypervirulent and multidrug-resistant Enterococci isolates leading to infections with adverse clinical outcomes could emerge. Zou et al. examined the correlation between erythromycin resistance and virulence genes and found a positive association between the presence of *gelE* and resistance to erythromycin [21]. This study was therefore conducted to examine clinical isolates of *E. faecalis* and *E. faecium* in Kenya to understand the prevalence and distribution of the virulence genes and determine if there is an association between antimicrobial resistance, clinical presentation, demographic factors, and virulence genes.

## 2. Methods

### 2.1. Study Design and Population

This cross-sectional study is nested in a multi-hospital surveillance study where patients over two months old with UTIs or SSTIs were recruited between May 2015 and December 2019 from six hospitals in five Kenyan counties. Urine samples, pus, and swabs from soft tissue infections were collected from study subjects after they consented to participate in the study. In addition, demographic and clinical data were collected for each patient, including gender, age, infection type, in- or outpatient status, infection acquisition in the community or hospital, and immune status.

### 2.2. Bacterial Isolation and Identification

Samples were shipped to the Kenya Medical Research Institute (KEMRI) laboratories at 2–8°C for pus and wound swabs and at room temperature for urine samples in boric acid. All samples were inoculated on MacConkey (BD, New Jersey, United States of America), cysteine lactose electrolyte deficient agar (HIMEDIA, Mumbai, India), and sheep blood agar (SBA) and incubated for 24 to 48 hrs at 37°C. Colony morphology and culture characteristics were observed macroscopically. Preliminary identification of Enterococci was made based on observation of Gram-positive cocci in chains on Gram stain and a negative catalase test. Confirmation of the identification and *Enterococcus* speciation were performed on the VITEK 2 automated platform (bioMérieux, Marcy-l'Étoile, France).

### 2.3. Antimicrobial Susceptibility Testing

Antimicrobial susceptibility tests were performed on the VITEK2 (bioMérieux, Marcy-l'Étoile, France) platform using the AST-P586 card panel consisting of thirteen antibiotics: benzylpenicillin, ampicillin, gentamicin, streptomycin, levofloxacin, erythromycin, quinupristin/dalfopristin, linezolid, vancomycin, teicoplanin, tetracycline, tigecycline, and nitrofurantoin. Results were interpreted based on Clinical & Laboratory Standards Institute (CLSI 2018) guidelines and the VITEK2 advanced expert system.

### 2.4. Detection of Virulence Genes

DNA was extracted from pure *Enterococcus* spp. isolate cultures using the Quick-DNA/Fungal/Bacterial extraction kit (ZymoResearch, California, USA) according to the manufacturer's instructions and quantified using the Nanodrop (Thermofisher, Massachusetts, USA) spectrophotometer. A multiplex PCR targeting the five genes (*cylA, asa1, gelE, esp, and hyl*) was performed on Applied Biosystems 9700 thermocycler (Thermofisher, Massachusetts, USA) using published primers by Vankerkhoven et al. 2004 [13]. Each 25 *μ*l of the PCR mixture consisted of 12 *μ*l of Dream Taq DNA polymerase (Thermofisher, Massachusetts, USA). 2.5 *μ*l of DNA, 0.1 *μ*l of *cylA*, asa1, *gelE*, and *hyl*. 0.2 *μ*l of *esp* specific primers [13]. The positive and negative controls were *E. faecalis* ATCC 29212 and *E. coli* ATCC 25922, respectively. The PCR conditions were: initial activation at 95°C for 15 min, 30 cycles of denaturation at 94°C for 1 min, annealing at 56°C for 1 min, extension at 72°C for 1 min, and finally, one extension cycle at 72°C for 7 mins. The PCR products were observed by running 15 *μ*l of the PCR reaction on a 1% agarose gel alongside a 100 bp ladder (Thermofisher, Massachusetts, United States) and visualizing on an E-box gel documentation station (Vilber, Marne-la-Vallée, France).

## 3. Data Analysis

Demographic and clinical data were extracted from study questionnaires and displayed in an Excel spreadsheet. Quantitative data were analyzed in Excel. The association between virulence genes, demographic factors, and antimicrobial susceptibility phenotypes was assessed using the Fisher's exact test with a *P* ≤ 0.05 considered significant.

## 4. Results

### 4.1. Demographic and Clinical Characteristics of Patients with Enterococcal Infections

*Enterococcus* isolates were obtained from six hospitals within five counties: Nairobi (16), Kisumu (18), Kericho (2), Kisii (7), and Kilifi (1). Thirty-seven *E. faecalis* isolates and 7 *E. faecium* isolates were isolated from forty-four patients with a mean age of 37 years during the study period which represented only 1.89% of the total isolates obtained during the same period. The majority of isolates 29/44 (65.1%) were from the skin and soft tissue infections (SSTIs) while 15/44 (34.9%) were isolated from urinary tract infections (UTIs). Ten out of forty-four patients (23%) were immunocompromised individuals, 28/44 (63.7%) were hospitalized, and 10/44 (23.0%) had hospital-associated infections. *E. faecalis* was isolated more from females 21 (56.7%) than males. In contrast, 6 of the 7 *E. faecium* isolates were from males. All seven *E. faecium* isolates were from hospitalized patients and 6/7 from patients with SSTIs. Among the 44 patients, most had community-acquired enterococci infections, but *E. faecium* infections accounted for most of the hospital-associated infections. Only one UTI was hospital-associated compared with six SSTIs (Table 1).

Age was significantly associated with Enterococci infections (*P*=0.0270). The age group between 14 and 29 years contributed the largest proportion of infections, followed by the age group between 31 and 49 years and above 50 years. There was no association between gender and the species of *Enterococcus* or between immunocompromised status or type of infection with enterococcal species. There was a significant association between species type, the source of infections (*P*=0.0367), and the patient status (inpatient/out-patient). Community-acquired Enterococci infections were more likely to be caused by *E. faecalis* than *E. faecium* whereas *E. faecalis* were more likely to be HAI with all *E. faecalis* infections identified in hospitalized patients (Table 1).

### 4.2. Phenotypic Characteristics and Antimicrobial Susceptibility Profiles of the Enterococci spp. Isolates

**Figure 1:**
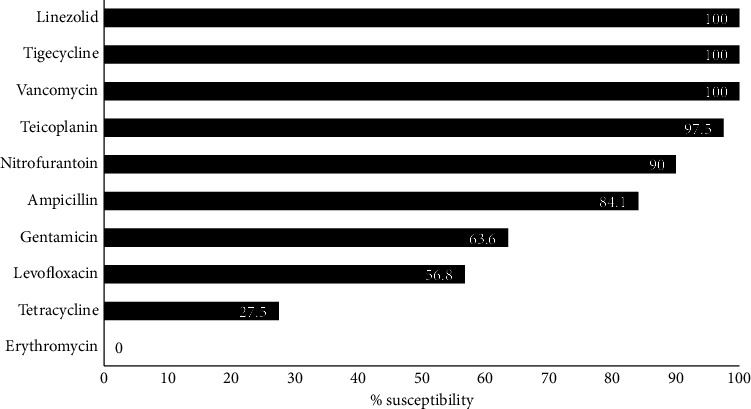
Antimicrobial susceptibility profile of *Enterococcus* spp. isolates (*n* = 44).

All 37 *E. faecalis* and 7 *E. faecium* colonies appeared as smooth, nonhemolytic yellow colonies with entire edges on CLED media indicating lactose fermentation. They were all Gram-positive cocci in short chains microscopically. Among the 44 isolates, there was 100% susceptibility to three antibiotics (linezolid, tigecycline, and vancomycin). There was 97.5%, 90.0%, 84.1%, 63.6%, 56.8%, and 27.5% susceptibility to teicoplanin, nitrofurantoin, ampicillin, gentamicin, levofloxacin, and tetracycline, respectively. Complete resistance to erythromycin was also observed (Figure 1). Supplementary Table 1 contains raw data of phenotypic antibiotic resistance data.

### 4.3. Detection of Virulence Genes

**Figure 2:**
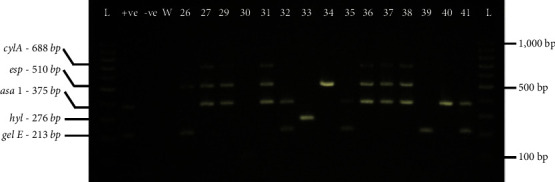
A representative gel electrophoresis image indicating the presence or absence of the five virulence genes in some of the *Enterococcus* spp. isolates. L-100 bp ladder; +ve-positive control *E. faecalis* ATCC 29212; −ve-negative control *E. coli* ATCC 25922; W-nuclease-free water; Lane numbers-individual isolates; bp-base pairs.

All the five virulence genes screened were detected among the *E. faecalis* and *E. faecium* isolates based on the presence of expected band sizes (Figure 2). Data indicating the presence or absence of the virulence genes for all the isolates is shown in Supplementary Table 1.

*gelE* was the most frequently detected gene at 27 (61.4%) followed by *asa1* 26 (59.1%), *esp* 16 (36.3%), *cylA* 11 (25.0%) and *hyl* 1 (2.3%) (Table 2). All the *asa1* and *cylA* genes detected were in *E. faecalis*. *gelE* gene was detected in 26/27 (96.3%) *E. faecalis* isolates. 9/37 (24.3%) of *E. faecalis* isolates had only one of the virulence genes, 15/37 (40.54%) had two genes, 10/37 (27.03%) had three genes, and 2/37 (5.45%) had four genes. The most common gene combinations were *gelE* and *asaI* (35.1%) followed by *asa1, cylA, and esp* at 13/44 (24.3%) both found only in *E. faecalis. E. faecium* carried fewer virulence genes than *E. faecalis,* with 4/7 (57.0%) having none of the genes, two isolates having only one gene, and a single isolate having two genes (*gelE,* and *esp*). Notably, *hyl* was only detected in an *E. faecium* isolate. In contrast, *cylA* and *asa1* were only detected in *E. faecalis*.

### 4.4. Distribution and Association of Virulence Factors with Antibiotic Resistance

Data on virulence factors and antibiotic resistance are summarized in Supplementary Table 1. There was a significant association between tetracycline resistance and the presence of *asa1* and *esp* (*P*=0.0305 and 0.0363, respectively), nitrofurantoin resistance and the presence of *gel E* and *asa1* genes (*P*=0.0175 and 0.0225, respectively) and ampicillin resistance and the presence of *gel E* and *asa1* (*P*=0.0005 and 0.0008, respectively) (Table 3). The associations between the other antibiotics were not tested because of complete or almost complete resistance or susceptibility to erythromycin, teicoplanin, nitrofurantoin, linezolid, vancomycin, and tigecycline.

## 5. Discussion

This study was conducted to evaluate the prevalence and distribution of known *Enterococcus* spp. virulence genes among clinical *E. faecalis and E. faecium* isolates obtained in a 5-year antimicrobial resistance surveillance study. The study found that *Enterococcus* spp. are uncommon clinical pathogens in the sampled Kenyan population given that the *E. faecalis* and *E. faecium* isolates represented <2% of the isolates recovered from clinical samples in the parent study. This is consistent with previous research in Africa that showed a 2.7% prevalence of *Enterococcus* spp. infections among pediatrics [22] much lower compared to a 13.6% prevalence reported in East Asia. The isolates in this study were obtained from SSTIs and UTIs consistent with the known infections caused by Enterococci spp. [23]. However, we observed that *E. faecalis* was the predominant pathogen of the two species with *E. faecalis* isolated five times more than *E. faecium*. *E. faecalis*'*s* dominance is consistent with studies by Goel et al. in North India [24], that identified *E. faecalis* as the main uropathogen in community-acquired UTIs (CA-UTIs). This abundance of *E. faecalis* in CA-UTI could be attributed to the predominance of *E. faecalis* in the patient's commensal flora [25, 26]. *E. faecium* though less frequently isolated, was found predominantly in male hospitalized patients with SSTIs, indicating its significance as a hospital-associated pathogen compared to other *Enterococcus* species.

Despite having fewer of the virulence genes tested than *E. faecalis, E. faecium* infections were associated with inpatient infections which implies infections of greater severity. This apparent disparity was also reported by Higuita et al. [27], who observed that *E. faecium* caused more severe infections and had a higher mortality rate than *E. faecalis*. Based on our observations we hypothesize that hospital-associated *E. faecium* infections could be opportunistic, affecting already vulnerable patients and leading to more adverse outcomes, and could have little to do with the presence or absence of virulence factors.

When we examined the antibiotic resistance patterns we found no significant differences between the two species in contrast to studies that found that *E. faecium* isolates are more efficient in accumulating resistance genes [6], resulting in greater resistance to penicillin, ampicillin, piperacillin, imipenem, and ciprofloxacin than *E. faecalis* isolates The lack of difference in resistance between species could reflect the rarity of human enterococcal infections and the high number of community-acquired infections which do not experience the antibiotic pressure that would drive antibiotic resistance to drugs typically used in hospital settings. The AST results indicated many available treatment options for the clinical management of Enterococcal infections since all the isolates tested were susceptible to vancomycin, tigecycline, and linezolid. The samples also had high susceptibility to teicoplanin, nitrofurantoin, ampicillin, gentamycin, and levofloxacin. Variable *E. faecalis* antibiotic resistance results for rifampin (60.7%), tetracycline (17.9%), erythromycin (14.3%), and chloramphenicol (10.7%) have been documented in Kenya [27]. A high incidence of antibiotic resistance to ampicillin (80%) and doxycycline (73.3%) have also been reported in Ethiopia [18].

Glycopeptide resistance which is mediated by the *Van* gene clusters has rarely been reported in studies from Kenya [28, 29] and we did not observe any resistance in this study. Enterococci expressing the *vanA* gene are highly resistant to vancomycin and teicoplanin antibiotics, while enterococci expressing the *vanB* gene show high resistance to vancomycin and susceptibility to teicoplanin [30]. In this study, there was no resistance to vancomycin, but teicoplanin nonsusceptible isolates were observed in a few isolates and confirmed by repeat testing. Discordant resistance to the glycopeptide antibiotics is a rare but reported occurrence attributed in a study by Loong et al. [31] to novel point mutations and deletions in the regulatory regions for the Van genes located in the Tn1546 type transposon. Analysis of the presence of vancomycin resistance genes and the regulatory components could offer clues on the mechanisms at play in the unusual discordance observed among the study isolates.

The study observed high susceptibility (75%) to nitrofurantoin, the second most common drug to treat bacterial infections in the urinary tract in Kenya after beta-lactam drugs [28]. This high susceptibility in Kenya compared to other countries such as Iran with resistance levels of 35.5% [32] suggests that nitrofurantoin use is still not as common in Kenya as in other countries. Erythromycin is a relatively inexpensive broad-spectrum antibiotic used to treat many infections, so it was not surprising that resistance levels were high. This study shows that this drug is no longer effective for the treatment of *Enterococcus* spp. infections. A study in Nigeria also showed 100% erythromycin resistance, indicating that this trend is prevalent in more than one region of sub-Saharan Africa [33]. This study on clinical isolates found tetracycline resistance levels (72.5%) markedly higher than the 17.9% percent reported in the 2018 survey by Wambui et al. performed on slaughterhouse cattle [27] but consistent with studies done in a Kenyan hospital in 2009 that found tetracycline resistance rates of 80% for *E. faecalis* and 71% for *E. faecium* [34]. These high rates can be attributed to tetracycline being commonly used to treat infections because it is affordable and readily available. The high tetracycline resistance rate has also been linked to the widespread use of tetracycline in livestock [27], evidenced by its presence in animal products [35, 36] and among poultry in Europe [37] which could drive antibiotic resistance in bacteria including Enterococci.

This study reports gentamicin resistance of 36.4%, which suggests close to a two-fold increase in resistance in less than ten years based on a reported 19% resistance level in Kenya in 2012 [34]. Since aminoglycosides (e.g., gentamicin) monotherapy is known to have poor uptake into the cytoplasm, a combination of penicillin/gentamicin therapy is recommended for the treatment of patients with Enterococcal infections to improve the penetration of gentamicin through the bacterial cell-wall using the cell-wall active penicillins, resulting in synergistic activity [38]. A study done in Kenya in 2020 by Maina et al. [39] reported that combinations of penicillin and gentamicin were predominant in the neonatal unit (58%). Resistance to ampicillin was roughly comparable to resistance to gentamycin in this study, implying corresponding use.

After observing that the antibiotic resistance levels were low except for erythromycin and tetracycline we considered whether the pathogenicity of the organisms were a significant threat by examining the virulence gene profiles. The gene encoding gelatinase *gelE* was the most prevalent virulence gene (36.3%). This is consistent with the findings of numerous studies conducted around the world, all of which found *gelE* to be more prevalent than the other genes measured in this study [40]. In terms of dominance, the agglutination substance gene *asa1* came in second. Both genes were present only in *E. faecalis*. *gelE* and *asa1* are the predominant virulence genes in similar studies performed among clinical isolates in India and Iran and also in pigs from a study in China [21, 40, 41]. The *hyl* gene was found in only one *E. faecium* isolate, which was not a surprise given earlier studies that observed that the *hyl* gene was predominantly found in *E. faecium* isolates from clinical samples in the United States [6]. Although *esp* is known for its function in adhesion to the urinary tract wall [12] in this study, it was equally present in isolates from UTIs and SSTIs.

The study also sought to test the association of virulence genes with antibiotic resistance. In addition to the association described by Zou et al. between *gel E* and erythromycin, this study has identified other significant associations between the *asa1* gene and *esp* and tetracycline and between nitrofurantoin, *gelE*, and *asa1*. The *asa1*, *gelE*, and *esp* genes encode biofilm-forming proteins involved in adhesion to the host cells [8, 42]. The positive association between *asa1*, *gelE*, and *esp* genes with nitrofurantoin and tetracycline resistance is interesting as the two drugs are effective against biofilm-forming isolates [43, 44]. Biofilm-forming Enterococci bacteria are generally more resistant to antibiotics than nonbiofilm-forming ones [45]. We hypothesize that exposure of biofilm-forming isolates to nitrofurantoin and tetracycline could drive specific resistance to these agents. The association of *asa1*, *esp,* and *gelE* in antibiotic-resistant bacteria is advantageous as it provides a double arsenal for causing and surviving the treatment of clinical infections.

The study had a few limitations. First, there were only a few *Enterococcus* spp. isolates obtained in the study, which limited the statistical analysis and inferences that could be made. Second, some patients had coinfections with other pathogens, so the infections could not be solely attributed to *Enterococcus* spp. Third, the study only screened for five essential virulence genes, whereas more genes are involved in Enterococcal pathogenicity. Fourth, although most of the virulence genes tested are associated with biofilm formation, biofilm assays were not conducted to confirm the phenotype. A more extensive study combining phenotypic assays and whole-genome approaches would address virulence factors more comprehensively and provide the isolates' strain types to address any clonality issues that could skew the detected associations.

## 6. Conclusion

The study has identified *E. faecium* as a predominantly health-care-associated infection affecting male patients and *E. faecalis* as an important etiology of community-acquired urinary tract infections. Enterococcal infections can be well managed due to the low-level resistance observed for most antibiotics except tetracycline and erythromycin. All five virulence genes tested were identified among the Kenyan isolates with *E. faecalis* carrying more and multiple genes. The importance of *esp, asa1,* and *gelE* virulence genes in virulence and their co-occurrence with antibiotic resistance could explain the clinical success of *E. faecalis* and provides an opportunity for further research.

## Figures and Tables

**Table 1:** Demographic and clinical characteristics of patients with *E. faecalis*/*faecium* infections.

Variables		*E. faecalis*	*E. faecium*	*P* value
Gender	Male	17	6	0.0973
	Female	20	1	

Age	1–13 years	2	0	**0.0270**
	14–29 years	13	2	
	30–49 years	11	3	
	≥50 years	11	2	

Admission status	Inpatient	21	7	**0.0370**
	Outpatient	16	0	

Immunocompromised	Yes	8	2	0.6490
	No	29	5	

Source of infection	HAI	6	4	**0.0367**
	CAI	31	3	

Infection type	SSTI	23	6	0.3926
	UTI	14	1	

Bold-Fisher's exact test significance of *P* ≤ 0.05. HAI, hospital-associated infections; CAI, community-acquired infections; SSTIs, skin and soft tissue infections; UTIs, urinary tract infections.

**Table 2:** Frequency and profiles of virulence genes for *E. faecalis* and *E. faecium* isolates.

	*E. faecalis* (*n* = 37)	*E. faecium* (*n* = 7)	All isolates (*n* = 44)
	*n* (%)	*n* (%)	*n* (%)
*Frequency of virulence genes*			
*Esp*	14 (87.5)	2 (12.5)	16 (36.3)
*hyl*	0 (0)	1 (100)	1 (2.27)
*asa1*	26 (100)	0 (0)	26 (59.1)
*gel E*	26 (96.3)	1 (3.7)	27 (61.4)
*cyl A*	11 (100)	0 (0)	11 (25.0)
*None*	1 (20)	4 (80)	5 (11.4)

*Virulence gene profiles*			
No genes	1 (2.7)	4 (57.1)	5 (11.3)
*gel E* only	8 (21.6)	0 (0)	8 (18.2)
*asa1* only	1 (2.70)	0 (0)	1 (2.3)
*hyl* only	0 (0)	1 (14.2)	1 (2.3)
*esp* only	0 (0)	1 (14.2)	1 (2.3)
*gel E, esp*	2 (5.4)	1 (14.2)	3 (6.8)
*gel E, asa1*	13 (35.1)	0 (0)	13 (29.6)
*gel E, asa1, esp*	1 (2.7)	0 (0)	1 (2.3)
*asa1, cyl A, esp*	9 (24.3)	0 (0)	9 (20.5)
*gel E*, *asa1*, *cyl A*, *esp*	2 (5.4)	0 (0)	2 (4.6)

**Table 3:** Association of resistance to antibiotics and virulence factors.

	Tetracycline			Nitrofurantoin			Levofloxacin			Gentamicin			Ampicillin		
	*R*/*I*	*S*	*P* value	*R*/*I*	*S*	*P* value	*R*/*I*	*S*	*P* value	*R*/*I*	*S*	*P* value	*R*/*I*	*S*	*P* value
*gel E* (+ve)	18	9	0.1585	0	27	**0.0175**	**9**	**18**	0.1252	**6**	**21**	0.1067	**0**	**27**	**0.0005**
*gel E* (−ve)	15	2		4	13		10	7		8	9		7	10	

*asa1* (+ve)	23	3	**0.0305**	0	26	**0.0225**	**12**	**14**	0.7600	**11**	**15**	0.3607	**0**	**26**	**0.0008**
*asa1* (−ve)	10	8		4	14		7	11		5	13		7	11	

*cyl A* (+ve)	11	1	0.239	0	11	0.5579	4	7	0.7315	5	6	0.4921	0	11	0.1652
*cyl A* (−ve)	22	10		4	28		15	18		11	22		7	26	

*esp* (+ve)	15	1	**0.0363**	1	14	1	6	10	0.753	8	8	0.2002	1	15	0.3930
*esp* (−ve)	18	10		3	29		13	15		8	20		6	22	

*hyl* (+ve)	1	0	0.222	1	0	0.0909	0	1	1	0	1	1	1	0	0.1591
*hyl* (−ve)	32	11		3	40		19	24		14	29		6	37	

*R*, resistant; *I*, intermediate; *S*, susceptible. Bold-Fisher's exact test significance at *P* ≤ 0.05.

## Data Availability

The graphs, figures, and tables data used to support the findings of this study are included within the article and also uploaded in the figures files section and supplemental files section.

## Abbreviations

asa1: Agglutination substance
ATCC: American Type Culture Collection
CAI: Community-acquired infection
CLED: Cysteine lactose electrolyte deficient agar
CLSI: Clinical & Laboratory Standards Institute
cylA: Cytolysin A
DNA: Deoxyribonucleic acid
*E. faecalis*: *Enterococcus faecalis*
*E. faecium*: *Enterococcus faecium*
esp: Enterococcal surface protein
*gelE*: *Gelatinase*
HAI: Hospital-associated infection
*hyl*: Hyaluronidase
IRB: Institutional Review Board
KEMRI: Kenya Medical Research Institute
MLST: Multilocus sequence typing
PCR: Polymerase chain reaction
SBA: Sheep blood agar
SERU: Scientific and Ethics Review Unit
SSTIs: Skin and soft tissue infections
UTIs: Urinary tract infections
WRAIR: Walter Reed Army Institute of Research.

## Supplementary Materials

Supplementary Materials Supplementary Table 1: infection types, antibiotic resistance profiles, and virulence genes for the *Enterococcus* spp. isolates.
