# Supplementary material for: Determination of Enterococcus faecalis and Enterococcus faecium Antimicrobial Resistance and Virulence Factors and Their Association with Clinical and Demographic Factors in Kenya
Source: J Pathog. 2022 Nov 9;2022:3129439. doi: 10.1155/2022/3129439 (PMC9668473; doi:10.1155/2022/3129439)
Supplement: Supplementary Materials — Supplementary Table 1: infection types, antibiotic resistance profiles, and virulence genes for the Enterococcus spp. isolates. [file 3129439.f1.docx]

**Supplementary Table 1.** Infection types, antibiotic resistance profiles, and virulence genes for the *Enterococcus* spp. isolates

| **Isolate code** | **Enterococcus spp.** | **Source county** | **Infection type** | **Antibiotics** | | | | | | | | | | | | **Virulence genes** | | | | |
| --- | --- | --- | --- | --- | --- | --- | --- | --- | --- | --- | --- | --- | --- | --- | --- | --- | --- | --- | --- | --- |
|  |  |  |  | ERY | TET | NIT | TEC | | LNZ | | LEV | GEN | AMP | VAN | TIG | *gel E* | *asa1* | *cyl A* | *esp* | *hyl* |
| EF4 | *E. faecalis* | Nairobi | UTI | **R** | **R** | S | S | S | | **R** | | S | S | S | S | X |  |  |  |  |
| EF2 | *E. faecalis* | Nairobi | UTI | **R** | **R** | S | S | S | | **R** | | **R** | S | S | S | X | X |  |  |  |
| EF15 | *E. faecalis* | Nairobi | UTI | **R** | S | S | S | S | | S | | S | S | S | S | X | X |  |  |  |
| EF21 | *E. faecalis* | Kisumu | UTI | **R*** | **R** | S | S | S | | I | | S | S | S | S | X | X |  |  |  |
| EF22 | *E. faecalis* | Kisumu | UTI | **R** | **R** | S | S | S | | I | | S | S | S | S | X | X |  |  |  |
| EF23 | *E. faecalis* | Kisumu | UTI | **R** | **R** | S | S | S | | I | | S | S | S | S | X | X |  |  |  |
| EF24 | *E. faecalis* | Kisumu | UTI | **R** | **R** | S | S | S | | S | | S | S | S | S | X | X |  |  |  |
| EF38 | *E. faecalis* | Kisumu | UTI | **R** | **R** | S | S | S | | S | | **R** | S | S | S |  | X | X | X |  |
| EF13 | *E. faecalis* | Nairobi | UTI | **R** | **R** | S | S | S | | I | | S | S | S | S |  | X | X | X |  |
| EF17 | *E. faecalis* | Nairobi | UTI | **R** | **R** | S | S | S | | **R** | | S | S | S | S |  | X | X | X |  |
| EF20 | *E. faecalis* | Kericho | UTI | **R** | **R** | S | S | S | | S | | S | S | S | S | X | X | X | X |  |
| EF19 | *E. faecalis* | Kericho | UTI | **R** | **R** | S | S | S | | S | | S | S | S | S | X |  |  | X |  |
| EF27 | *E. faecalis* | Kisumu | UTI | **R** | **R** | S | S | S | | S | | S | S | S | S |  | X | X | X |  |
| EF29 | *E. faecalis* | Kisumu | UTI | **R** | **R** | S | S | S | | I | | **R** | S | S | S |  | X | X | X |  |
| EF28 | *E. faecalis* | Kisumu | SSTI | **R** | **R** | S | S | S | | I | | **R** | R | S | S |  |  |  |  |  |
| EF5 | *E. faecalis* | Nairobi | SSTI | **R** | S | S | S | S | | S | | S | S | S | S | X |  |  |  |  |
| EF6 | *E. faecalis* | Nairobi | SSTI | **R** | S | S | S | S | | S | | S | S | S | S | X |  |  |  |  |
| EF9 | *E. faecalis* | Nairobi | SSTI | **R** | S | S | S | S | | S | | S | S | S | S | X |  |  |  |  |
| EF10 | *E. faecalis* | Nairobi | SSTI | **R** | S | S | S | S | | S | | S | S | S | S | X |  |  |  |  |
| EF12 | *E. faecalis* | Nairobi | SSTI | **R*** | S | S | **I** | S | | S | | **R** | S | S | S | X |  |  |  |  |
| EF46 | *E. faecalis* | Kilifi | SSTI | **R** | S | S | S | S | | S | | S | S | S | S | X |  |  |  |  |
| EF39 | *E. faecalis* | Kisii | SSTI | **R** | S | S | S | S | | S | | S | S | S | S | X |  |  |  |  |
| EF3 | *E. faecalis* | Nairobi | SSTI | **R** | **R** | S | S | S | | S | | S | S | S | S | X | X |  |  |  |
| EF16 | *E. faecalis* | Nairobi | SSTI | **R** | **R** | S | S | S | | **R** | | **R** | S | S | S | X | X |  |  |  |
| EF14 | *E. faecalis* | Nairobi | SSTI | **R** | **R** | S | S | S | | **R** | | **R** | S | S | S | X | X |  |  |  |
| EF25 | *E. faecalis* | Kisumu | SSTI | **R*** | **R** | S | S | S | | S | | S | S | S | S | X | X |  |  |  |
| EF32 | *E. faecalis* | Kisumu | SSTI | **R** | **R** | S | S | S | | **R** | | **R** | S | S | S | X | X |  |  |  |
| EF41 | *E. faecalis* | Kisii | SSTI | **R** | S | S | S | S | | S | | S | S | S | S | X | X |  |  |  |
| EF35 | *E. faecalis* | Kisumu | SSTI | **R** | **R** | S | S | S | | S | | S | S | S | S | X | X |  |  |  |
| EF26 | *E. faecalis* | Kisumu | SSTI | **R*** | **R** | S | **I** | S | | S | | S | S | S | S | X |  |  | X |  |
| EF45 | *E. faecalis* | Kisii | SSTI | **R** | **R** | S | S | S | | **R** | | **R** | S | S | S | X | X |  | X |  |
| EF44 | *E. faecalis* | Kisii | SSTI | **R** | **R** | S | S | S | | S | | **R** | S | S | S |  | X | X | X |  |
| EF31 | *E. faecalis* | Kisumu | SSTI | **R** | **R** | S | S | S | | I | | **R** | S | S | S |  | X | X | X |  |
| EF36 | *E. faecalis* | Kisumu | SSTI | **R** | **R** | S | S | S | | S | | S | S | S | S |  | X | X | X |  |
| EF37 | *E. faecalis* | Kisumu | SSTI | **R** | **R** | S | S | S | | S | | **R** | S | S | S |  | X | X | X |  |
| EF40 | *E. faecalis* | Kisii | SSTI | **R** | S | S | S | S | | S | | S | S | S | S |  | X |  |  |  |
| EF42 | *E. faecalis* | Kisii | SSTI | **R** | **R** | S | S | S | | S | | S | S | S | S | X | X | X | X |  |
| EF43 | *E. faecium* | Kisii | SSTI | **R*** | **R** | S | S | S | | S | | S | S | S | S | X |  |  | X |  |
| EF33 | *E. faecium* | Kisumu | SSTI | **R** | **R** | **I** | S | S | | S | | S | **R** | S | S |  |  |  |  | X |
| EF34 | *E. faecium* | Kisumu | SSTI | **R** | S | **R** | S | S | | **R** | | **R** | **R** | S | S |  |  |  | X |  |
| EF7 | *E. faecium* | Nairobi | SSTI | **R** | **R** | S | S | S | | **R** | | S | **R** | S | S |  |  |  |  |  |
| EF8 | *E. faecium* | Nairobi | SSTI | **R** | **R** | S | S | S | | **R** | | S | **R** | S | S |  |  |  |  |  |
| EF11 | *E. faecium* | Nairobi | SSTI | **R** | **R** | **I** | S | S | | I | | S | **R** | S | S |  |  |  |  |  |
| EF30 | *E. faecium* | Kisumu | UTI | **R** | **R** | **I** | S | S | | **R** | | **R** | **R** | S | S |  |  |  |  |  |

**R**- resistant; **R***- VITEK2 Advanced expert system interpreted resistance; S- susceptible; **I** - intermediate; ERY- Erythromycin; TET-Tetracycline; LNZ - Linezolid; TEC - Teicoplanin; VAN - Vancomycin; TIG - Tigecycline; AMP - Ampicillin; GEN – Gentamycin; LEV – Levofloxacin; NIT - Nitrofurantoin; X - the presence of the virulence genes
